# From Isolation to Integration: Implementing a Reliable Weekend Handover System in Acute Psychiatric Services

**DOI:** 10.1192/bjo.2026.11461

**Published:** 2026-06-30

**Authors:** Matthew Nelson, Helen Popov, Rebecca Gilmore, Emily Lowe, Abeku Mensah

**Affiliations:** Norfolk and Suffolk Foundation Trust, Norwich, United Kingdom

## Abstract

**Aims:**

Weekend medical handover across Central Norfolk acute psychiatric services has historically been inconsistent and poorly structured, contributing to delayed reviews, duplicated tasks, reduced situational awareness, and resident doctor isolation. This Quality Improvement Project aimed to introduce a standardised weekend handover system to improve clinical safety, timeliness of care, and on-call doctor experience. Objectives included establishing a reliable weekend handover meeting, improving information completeness,facilitating early identification of unwell patients, and reducing inappropriate tasks passed to out-of-hours teams.

**Methods:**

Using PDSA cycles, we implemented: (1) a 09:00 Saturday and Sunday multi-site Teams handover for Tier 1 and Tier 2 doctors; (2) pre-arranged invites and reminders; (3) a structured digital handover log covering admissions, unwell patients, investigations, and tasks; and (4) a centralised Teams channel for document access. Surveys at two timepoints assessed communication, clarity, task appropriateness, and perceived support. Clinical outcomes from 63 weekend admissions (March–May 2025) were compared pre-intervention and post-intervention, including time to first medical review, senior review, and medication administration.

**Results:**

Baseline survey responses (n=16) showed moderate satisfaction (mean ≈3), with good supervision (3.6) but poor perceived handover structure (2.3). Willingness to attend weekend handover was 56%. Qualitative feedback reported improved communication, reduced isolation, and better planning for the day. Post-intervention data demonstrated improvements in time to first medical review (10.7%), physical-health medication (10.2%), and psychotropic administration (33.0%). Time to senior face-to-face review increased (97.2%). The reasons for this deterioration are unclear and likely multifactorial. One interpretation is that collective discussion of all new admissions during morning handover increased Tier 1 doctors’ sense of support, reducing perceived need for immediate escalation. Indirectly, this may reflect improved resident doctor confidence and adequacy of remote senior input, though further exploration is required. Barriers included technological unfamiliarity, variable engagement, and inappropriate task shifting from weekday teams.

**Conclusion:**

A structured weekend handover system is both feasible and beneficial for improving communication, early clinical planning, and aspects of patient care. Importantly, qualitative feedback highlighted a strong positive impact on resident doctors’ wellbeing, with many reporting reduced isolation, improved support, and greater confidence in managing weekend workloads. Next steps include automated invites, refining the digital handover log, and establishing a routine senior weekend review pathway, with potential for wider trust adoption.

